# Translations and Extracts from Foreign Journals

**Published:** 1873-11

**Authors:** J. H. Etheridge

**Affiliations:** Rush Medical College, Chicago


ARTICLE VII.—*Translations and Extracts from Foreign Journals.* By Prof. J. H. ETHERIDGE, M.D., of Rush Medical College, Chicago.

The following article is taken from the *Gazette Hebdomadaire de Med. et de Chir.*, (Paris), 26th Sept., 1873.

*Decortication* (*Barking*) *of the Nose, in Cases of Elephantiasis of that Organ.* M. A. PONCET, Interne, Hotel Dieu de Lyon. Service of M. Ollier.

The skin and subcutaneous cellular tissues of the nose frequently become the seat of hypertrophic growths, bearing with them most disgraceful deformities, instances of which every one has seen. It is, indeed, certain noses in luxuriant vegetation which command attention by their inordinate development; they present, here and there, protuberances and depressions, accompanied by a purple or velvet tint, bordering on (wine) redness. The hypertrophy has a slow growth, but it increases every day; it is principally seen in drinkers. After a copious repast, with numerous libations, the face is animated; the skin of the face, and especially of the nose and checks, becomes red; the circulation there seems more active; then, after a few hours the color becomes normal. With some drinkers the same cause will produce the same effect, *congestion*, which, transient at first, eventually becomes continuous; it produces a genuine dilatation of the superficial vessels.

The skin and the subcutaneous tissue then begin to thicken; the sebacious follicles, in great numbers in this region, become indurated, inflame, and their inflammation works disorder in the nutrition of these tissues, a sort of chronic inflammation eventuating in elephantiasis of the nose, (resulting.)

The hypertrophy, which by preference occupies the movable part of the organ, does not always present the same characteristics: at one time it is uniform, that is to say, all points rise equally; at another time, it rises unequally at some points and produces tumors, bumps (bosselures—nodules?) of various forms.

These latter become, by their dimensions, sources of functional troubles, that is, they constantly hinder, in a variable degree, respiration, articulation, and the prehension of food; by their volume they hinder binocular vision, and thus induce strabismus. Add to the deformity these troubles, and an offensive discharge, and it is easy to understand the sort of deformity the surgeon is occupied with and attempts to cure.

Many of the cases eventuating in elephantiasis of the nose are greeted with a smile, when they come to demand a cure for this nasal hypertrophy, and one is content to recommend no more drinking (for the future.) The advice is assuredly very good, it is of utility for the future, but for the present moment it does not benefit the deformity.

It is in cases such as this, that M. Ollier practices *decortication* of the nose. This expression, happily chosen for the surgeon, answers, as we shall see, the account (description) of the operation. The patient being anæsthetized, he incises the skin and the thickened tissues on the dorsum of the nose, then, seizing one of the borders of the wound with forceps, he proceeds to their dissection, being very careful not to touch the cartilages and to preserve intact the fibrous tissues which unite them. He also completely preserves the fibro-cartilaginous framework, in order not to compromise the form and functions of the nose—and, if we may be permitted the comparison, he works upon (manipulates) this organ with bistoury and forceps as one would with the fingers remove the shell from a fresh walnut without breaking the covering —or would remove the bark of a branch without injuring the wood.

In the different decortications, made by M. Ollier, the operation offers no other peculiarity than a hemorrhage, resulting from the great vascularity of the organ removed. The use of sponges wet in ice-water, in staunching hemorrhage, allows the completion of the operation without a single risk.

With the finger introduced into the nostrils from time to time, as recommended by M. Ollier, one can easily be cognizant of the thickness of the tissues that he replaces, and of the integrity of the fibro-cartilaginous support, which above all things must be preserved.

The decortication finished, compresses, wet with ice-water, are held for a few moments against the bleeding surfaces; then the dressing is attended to. M. Ollier, in order to control all hemorrhage, applies directly to the wound, some picked up charpie saturated with a dilute solution of perchloride of iron, and places compresses over it, holding it all with a broad band. This first dressing should remain on many days; the charpie should be allowed to separate and fall off of itself, being raised up by suppuration; later, when there is no longer fear of hemorrhage, a simple dressing suffices. A hemorrhage may occur many days after the operation and be abundant enough to put the patient's life in danger, if it be not quickly checked. It is a fact, of the modifications that are observed in the elasticity and calibre of vessels of hypertrophied organs, that after a section their walls do not collapse as before (comme auparavant); they remain patulous, comparable in a measure to the supra hepatic (sus-hepatiques) veins after a section of the liver; furthermore, formed at the expense of tissue, rich in vessels, the proud flesh is itself very vascular, and, until cicatrization is complete, the surgeon should be on his guard against hemorrhage.

Cicatrization commences from the edges of the skin of the dorsum of the lobule of the nose, as well as from the skin of the nostrils; at the end of two or three months it is completed.

The writer then delineates one "observation" at length. The patient, aged 55 years, was a heavy drinker, taking three or four bottles of wine with his dinner; his enormous nose was the butt of ridicule, and in those regions he was called the "Father of France" on account of it. "Upon entering the Dieu Hospital, his face presented, in front and on his cheeks, voluminous buttons of acne. The skin of these regions, thickened, was of a violet red color, but the nose, first of all, captivated attention. Comparable to a potato, it was divided into three portions, a middle and two lateral. The skin of a lively red, of mammillated appearance, furrowed (marked) by bluish vesicles, was studded with large buttons of acne. The left part had the form and volume of a chestnut; attached to the middle part, it was movable, and the patient called it *the nut.* The right part was a little less voluminous, but possessed the same characteristics.

"The patient being chloroformed and kept in the upright position, in a high chair, the head turned back against the chest of an assistant, M. Ollier performed decortication. He incised the skin upon the median line, the incision extending from the lobule to the anterior border of the (proper) bone of the nose; he seized the cutaneous flap with the forceps and dissected out the fleshy masses as far as the sound skin. Their weight was forty grammes. His principal care during the operation was to not touch the different cartilages which give to the movable part of the nose its form and suppleness."

Repeated hemorrhages annoyed the surgeons, during the process of healing of this patient's nose, and a fatal result was, with great care, evaded. Eventually, recovery greeted the efforts made to cure.

"The nose now has a normal shape, its volume is the same as that of an ordinary nose; the tissue of the cicatrix is less supple than the adjoining skin, leading one to suppose that an operation had been performed."

### SKILL.

The sagacity and accuracy of diagnosis, which the French physicians exhibit, are greatly to be admired. The Paris correspondent of the *London Times and Gazette*, writing under Sept. 22nd, gives the following instance:

"A woman aged about 40 years was suffering from facial hemiplegia on the left side, accompanied by amaurosis of the right eye. The patient was subject to epileptic fits and the paralytic symptoms which first affected the left side were becoming manifest on the right side of the face when the patient was carried off by cholera, which she caught in the ward. During her lifetime, Dr. Ball (the attending physician) had diagnosed a tumor at the base of the brain over the petrous portion of the right temporal bone and compressing the nerves that pass over and through it. The death of the patient offered an opportunity for verifying the diagnosis. But this was not the only point of interest in the case, for, during the life of the patient, it was not sufficient to diagnose a tumor in the brain, but what the nature of that tumor was, and, in an able lecture by Dr. Ball on the subject, he referred to the almost insurmountable difficulties that beset the question. One of the means employed for arriving at anything like a correct diagnosis consists in the use of therapeutic agents. For instance, where syphilis is suspected, as it was in the case under notice, notwithstanding the protestations of the patient to the contrary, the usual anti-syphilitic treatment should be adopted. Accordingly Gibert's syrup (mercury and iodide of potassium) was given, and was followed by marked and rapid improvement in the state of the patient. But what added to the difficulty of the diagnosis was, the patient had no marks of syphilis on her body, and even at the autopsy Dr. Ball felt some hesitation in pronouncing the tumor to be of syphilitic origin, so much did it resemble in size and consistence a glioma; but a small spot of disease on the internal surface of one of the parietal bones removed all doubt in the matter."

—

### OZONE AND CHOLERA.

The *Berlin Klinische Wochenschrift*, No. 34, contains an article from Prof. von Pettenkofer, prompted by the recent outbreak of cholera in Europe, the last season. He shows how little is known of the connection between epidemic visitations and deficiencies of ozone in the air at such times. Many hold the position now, that deficiency of ozone is one of the first prerequisites to an epidemic outbreak. The learned Professor avers that we are not in possession of enough facts to settle this question, as all methods heretofore employed do not estimate accurately the amount of ozone contained in the atmosphere. Instruments used are ozonoscopes instead of ozonometers—they tell the presence but not the amount of ozone. The principal defect in observations made is, that no account has been made of the amount of air which in a given time comes in contact with the ozone papers. The Professor induced Dr. Wolfhuegel to make observations with the iodized paper in the open air and within doors. The result is very instructive. One thousand litres of air, out doors, constantly induced obvious reaction, while the air of the different rooms of the physiological laboratory of Munich (large—and quite uninhabited) did not induce the least reaction, even when 12,000 or 15,000 litres of air were brought in contact with the instrument. This fact is the more remarkable, as it is well known that air in rooms, although doors and windows are closed, is continually changing, if even only in a slight degree. "As we, therefore, live in our dwellings for the most part without ozone in the air, the smaller or greater quantity in the open air cannot exert any great direct influence on our well being." He does not, however, assert that the amount of ozone in the free atmosphere may not play an important part in the economy of nature; but we sadly need some method of showing with exactitude the variations in this amount.

—

### TACTUS ERUDITUS.

*L' Institut* for Sept. 17, 1873, contains the following, concerning the normal pulse, by Professor Bouillard, communicated at the Academy of Sciences:

"It is possible to distinguish four in place of two periods in the normal pulse, *i. e.*, a first pulsation, separated by a period of rest from a second pulsation, much more feeble than the first, and itself followed by a period of rest. The observation must be made on a person with a slow pulse of not more than forty, in order to become convinced of the truth of this statement. M. Bouley observed that while his colleague was making his statements he tried in vain to get this double pulsation on himself. M. Bouillard replied, that he could well believe this, for during thirty years he had never suspected the existence of this double pulsation, which can only be easily perceived in persons with slow pulse."

The following articles are reported in the *Gazette Hebdomadaire*, Oct. 3rd, 1873. They are extracts of papers read before the "Forty-Fifth Congress of Naturalists and Physicians of Germany, at Leipzig, for the year 1872."

*Carbonate of Ammonia and Uræmic Poison.* By PROFESSOR ROSENSTEIN.

[Condensed from a lengthy paper. The main features are old—one or two, however, have never before appeared in print. ED.]

"Carbonate of ammonia introduced into the system, produces the complex phenomena which are comparable to those of epilepsy or to certain uræmic accidents.

"The convulsions which this poison provokes are of cerebral origin, since they are not produced after separation of the cord from the brain; they are probably the direct result of the action of carbonate of ammonia upon the same elements of the encephalic centres, and not an excitation, transmitted by the great sympathetic or by the vagus, and reflected under the form of a convulsion.

"Morphine, chloroform, chloral, are without any action upon the progress of the poison.

"Unstriped muscles escape the convulsive action. Abortion has never been the consequence of a parallel intoxication in animals.

"Carbonate of ammonia is eliminated by the kidneys, and its action is very ephemeral and fugitive when these latter are intact. A small quantity is also eliminated from the lungs. According to Prof. Rosenstein the essential difference of action of carbonate ammonia and uræmic poison rests wholly in this fact: the former provokes only epileptiform phenomena, while the second may produce various accidents, coma, convulsions, delirium. In the cases where the uræmic poison produces epileptic attacks, or where one encounters carbonate ammonia in the blood, he should not accuse the latter, because its presence one is not always able to find in parallel cases, and because the quantity found in the blood of animals is by no means in proportion to the intensity of the epileptic phenomena.

"Finally, Prof. Rosenstein added that the accidents following vesical and prostatic affections and called *ammoniemia*, should be clearly separated from carbonate ammonia poisoning. Epileptic symptoms characteristic of the latter intoxication are absolutely wanting in *ammoniemia*."

—

BROMINE IN CROUP.

"Dr. Schuetz, of Prague, spoke of the use of bromine in croup. He has obtained, he said, excellent results by inhalations of this metalloide; he obtained uniform success with the following: 50 centigrammes of purified bromine, 50 centigrammes of bromide of potassium, in 90 grammes of water. Dr. Goltwald confirmed the truth of these assertions, and asserted that the diphtheritic (faucal) membranes lose, under the influence of this precious and novel agent, their consistence, and can be removed very easily. In support of the observations of these two authors, we may say that within the last year, during our residence in the Children's Hospital, we placed some diphtheritic membranes in a concentrated solution of bromine, and to our great surprise, we saw them dissolve in less than ten minutes. Their solubility is less rapid in the presence of bromide of potassium. They do not entirely disappear in the latter and bromine, sooner than an hour."

—

PNEUMONIA.

"Dr. Raczorowsky read a paper on the treatment of *acute uncomplicated pneumonia*, founded on pathological grounds, new, and otherwise sound. He considers pneumonia as an infectious disease, due to the introduction of a vegetable parasite into the larynx. He cites cases of epidemic pneumonias, and endeavors to prove that the disease commences in the larynx and thence extends to the bronchi along the mucous passages. The therapeutical indications involve the four following points: 1st, To expel the parasites, the originators of the disease, by the aid of vomiting, especially by the aid of tartar emetic. 2nd, To combat or moderate the local irritation. (This second indication is accomplished, according to his view, effectually, by means of morphine hypodermics, renewed every six or eight hours; this therapeutic measure, in calming distress, fulfills equally well the 3rd indication.) 3rd, To combat reflex phenomena. Lastly, tonics and appropriate regimen (wine, milk and soup) answer the fourth indication, according to the author—sustain the patient's strength."

